# The Myth of Care Coordination: Whether Professional Care Coordination Improved Older Adults’ Health Outcomes?

**DOI:** 10.1093/geroni/igab046.3741

**Published:** 2021-12-17

**Authors:** Kedong Ding

**Affiliations:** Case Western Reserve University, Cleveland, Ohio, United States

## Abstract

**Background:**

Current evidence on the effects of Care Coordination (CC) on older adults’ well-being and health service utilization is inconsistent. Previous studies are mostly limited to regional data and focus mostly on nurse-led CC instead of layperson Care Coordinators like family caregivers. This study explores the effects of having CC in a national sample of U.S. older adults and whether the coordinators’ professionalism impacts the effect of having CC on multidimensional health outcomes (Health outcomes were conceptualized as physical health, healthcare utilization, and care encounters).

**Methods:**

Data were from the 2016 and 2018 waves of the Health and Retirement Study (HRS) (n=1,372). Multivariate regression models were used to examine the effects of CC on multidimensional health outcomes in 2016 and the longitudinal effects of having CC. We also tested the effect of Care Coordinators’ professionalism on the multidimensional health outcomes. All models controlled for sociodemographic characteristics and health status.

**Results:**

Findings suggest that having CC doesn’t have a positive effect on older adults’ health outcomes. Having CC was associated with an increased number of acute diseases (β = 0.16, p < .001) and nonacute diseases (β = 0.21, p < .01) in longterm. The results regarding cross-sectional effects show that receiving care from a Coordinator was related to increased health service utilization. Participants with professional Care Coordinators were more likely to report receiving person-centered care (OR=1.60, p<.05).

**Conclusion:**

This study demonstrates the limited effects of CCs on older adults’ physical health outcomes, and emphasized the importance of care coordinators’ qualifications.

